# Hingeless Negative Linear Compression in the Mechanochromic Gold Complex [(C_6_F_5_Au)_2_(μ-1,4-diisocyanobenzene)]**

**DOI:** 10.1002/anie.201302825

**Published:** 2013-08-02

**Authors:** Christopher H Woodall, Christine M Beavers, Jeppe Christensen, Lauren E Hatcher, Mourad Intissar, Andrew Parlett, Simon J Teat, Christian Reber, Paul R Raithby

**Affiliations:** Department of ChemistryUniversity of Bath, Bath BA2 7AY (UK); Research Complex at Harwell, Rutherford Appleton LaboratoryHarwell, Didcot, Oxon OX11 0FA (UK); Advanced Light Source, Lawerence Berkeley National LaboratoryBerkeley, CA 947240(USA); Department de Chemie, Universite de MontrealMontreal, Quebec H3C 3J7 (Canada)

**Keywords:** crystallography, gold, high pressure, mechanochromism, negative linear compression

The compression of a crystalline material under hydrostatic pressure always results in a reduction in volume of the solid, with the observed reduction typically occurring in all crystallographic cell parameters.^1^ However, there are a number of exceptional materials that have been shown to expand in a specific direction upon hydrostatic compression while maintaining a positive volume compression. The phenomenon of uni-or biaxial expansion under compression is known as negative linear compressibility (NLC).^1^ Rare examples of NLC have attracted attention recently owing to the potential applications in a range of materials, including body armor, artificial muscle actuators, and pressure sensors.^2^

NLC may be quantified using isothermal compressibility, measured in TPa^−1^ and defined as the relative rate of change in length with respect to pressure (*K*_l_=−(*δ*(In *l*)/*δp*)_*T*_) which may be used to compare and contrast the compressibility of different materials.2d Typically a material will display a positive value of compressibility (+5–+50 TPa^−1^); however, in materials exhibiting NLC the values are negative in certain directions. The largest values of NLC have been reported in framework materials. The metallocyanide framework Ag_3_[Co(CN)_6_] exhibits extremely large NLC at low pressures (−76 TPa^−1^ from ambient (ambient herein refers to 20 K and 0.0001 GPa) to 0.19 GPa) before transforming to a new high-pressure phase that also displays NLC over a greater pressure range (−5.3 TPa^−1^),^1^ while KMn[Ag(CN)_2_]_3,_ a structural analogue of Ag_3_[Co(CN)_6_] has been shown to demonstrate strong persistent NLC (−12 TPa^−1^ from ambient to 2.2 GPa).2d More recently, “giant” NLC has been attributed to a simple zinc gold cyanate complex Zn[Au(CN)_2_]_2._ The compound displays a remarkably large NLC effect parallel to the crystallographic *c*-axis that is the largest of any other material ever reported (−42(5)TPa^−1^ between ambient and 1.8 GPa).^3^ In all these materials the observation of large NLC is attributed to specific structural motifs where covalent bonds act as hinges allowing the material to behave in a manner that mimics a wine-rack or trellis.^1^, 2b, d In molecular complexes, the largest NLC effect has been reported by Shepherd et al. in the spin crossover complex [Fe(dpp)_2_(NCS)_2_]⋅py (dpp=dipyrido[3,2-a:2′,3′-c]phenazine, py=pyridine).^4^ The complex employs a scissoring mechanism in which the angles between the two dpp ligands and the two NCS ligands widen upon compression by pivoting about the central iron atom, resulting in extreme negative linear compressibility (−10.3(20) TPa^−1^ from ambient to 2.2 GPa).^3^, ^4^ The wine-rack and scissoring mechanisms are similar except that in the former the pivot is about a covalent bond whereas in the latter it is about an atomic center.

Also of relevance to the discussion is the phenomenon of negative thermal expansion (NTE), which is a thermally driven analogue of NLC. It is exhibited by linear dumbbell molecules such as (*S*,*S*)-octa-3,5-diyn-2,7-diol, which has exceptionally large positive and negative anisotropic NTE.^5^ This phenomenon has been attributed to a combination of their linear geometry and to the presence of strong hydrogen bonds that are able to act as hinges between molecules that facilitate a pivot mechanism similar to that in the metallocyanate frameworks or metal complexes displaying NLC effects.^4^–^6^

[(C_6_F_5_Au)_2_(μ-1,4-diisocyanobenzene)] **1** (Scheme 1) belongs to a family of linear organoauric complexes, several of which display the extraordinary phenomenon of mechanochromism or the alteration of the emission wavelength of a material upon mechanical grinding.^7^ The shift in luminescent emission of the material upon grinding has been attributed to its rod-like molecular geometry and the absence of any strong intermolecular interactions between the molecules. Such a structure facilitates the sliding of layers of molecules over one another to form new gold–gold interactions resulting in a characteristic emission shift from an intra-ligand-localized π–π* excited state (*λ*_max_=415 nm) to a broad gold based emission (*λ*_max_=533 nm). The similarity between the molecular structures of (*S*,*S*)-octa-3,5-diyn-2,7-diol and [(C_6_F_5_Au)_2_(μ-1,4-diisocyanobenzene)] **1** suggests that the gold dimer merits investigation as a potential NLC or NTE material. We now report the results of the high pressure single crystal X-ray crystallographic and luminescence emission spectroscopic study on **1**.

**Scheme 1:**
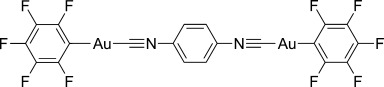
Molecular structure of [(C_6_F_5_Au)_2_(μ-1,4-diisocyanobenzene)] (1).

High-pressure single-crystal X-ray diffraction data was collected on crystals of **1** from ambient to 4.39 GPa at the Advanced Light Source synchrotron facility, Berkeley, California. Pressure was applied using a diamond anvil cell with methanol/ethanol as the hydrostatic medium and ruby fluorescence as the pressure calibrant.^8^ The unit cell volume decreases from 1002.5(2) Å^3^ at ambient pressure to 803.0(3) Å^3^ at 4.39 GPa. Fitting of a Birch Murnaghan third-order equation of state^9^ gives a bulk modulus of 7.5(7) GPa indicating that the material is “soft” for a crystalline material but not atypical for a organometallic complex.^10^

Analysis of the unit cell parameters reveals that while the *b* and *c* axes compress, as expected, with increasing pressure the *a* axis displays a large expansion of 0.2249(13) Å between ambient pressure and 2.42 GPa, an increase of 4.12 % of its original length (Figure 1). Calculation of the principal axes of compression and their relation to the monoclinic crystallographic axes reveals a net negative linear compression of −4.16 TPa^−1^ along the *a* axis across the entire pressure range studied (Supporting Information, Figure S4). If the data up to maximum length of the *a* axis at 2.42 GPa is considered, the negative linear compressibility is −12.57 TPa^−1^, making the NLC behavior similar in magnitude and duration to the extreme NLC displayed by KMn[Ag(CN)_2_]_3_ and [Fe(dpp)_2_(NCS)_2_].py.2d, 4 Above 2.42 GPa the NLC effect reduces as the crystal structure adopts more normal behavior under high pressure, but the *a* axes length remains longer than that observed at ambient pressure over the whole pressure range to 4.39 GPa.

**Figure 1:**
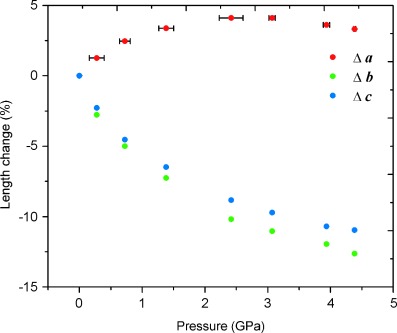
Change in unit cell length with pressure as a percentage of ambient length.

The simple rod-like molecular structure of **1** facilitates a direct correlation between the NLC behavior and molecular movement within the crystal by comparison of the crystal structures over a range of pressures. The ambient pressure structure has been reported previously;7a however, revisiting significant structural details is important to understand the interesting behavior. Each molecule sits on an inversion center positioned within the central bridging arene ring and the two gold atoms display a linear geometry (Scheme 1; Figure 2). There is an absence of strong intermolecular interactions throughout the structure; however, associative π–π interactions between adjacent the arene rings and the CN group (3.554(7) Å at ambient pressure to 2.97(3) Å at 4.39 GPa) lead to the association of the molecules into pairs which then aggregate into layers in the *ab* plane. These layers stack perpendicular to the *c* axis with molecules in each staggered in orientation relative to the plane above and below (Figure 2). The layers are separated by 9.419(2) Å at ambient pressure, and they are therefore independent of one another.

**Figure 2:**
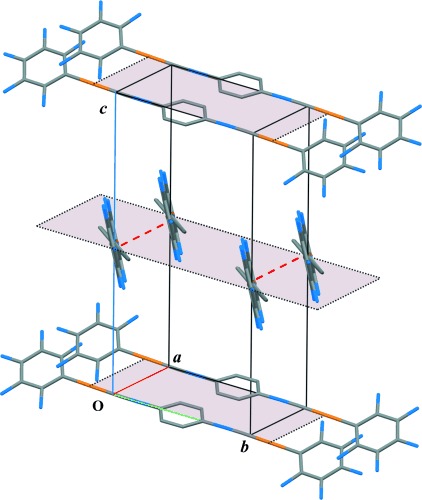
Molecular packing in the unit cell of [(C_6_F_5_Au)_2_(μ-1,4-diisocyanobenzene)] (1). The presence of associative π–π interactions are indicated with red dashes. The packing planes are marked for clarity.

The staggered arrangement between layers may be measured by the torsion angle through the gold atoms (Figure 3 a). Under ambient conditions, an angle of 74.314(9)° is observed between molecules, as pressure is increased the angle decreases to 63.87(18)° at 2.42 GPa, decreasing further to 63.14(3)° at 4.39 GPa. If viewed directly down the *c*-axis (Figure 3 b), it is clear that the molecular structure is reminiscent of the wine-rack or trellis motif attributed to the NLC in materials discussed earlier, with the reduction of the torsion angle with pressure effectively “closing the rack”. It is also clear that the closing process causes the expansion in the *a*-axis direction as the linear molecules orient themselves more nearly parallel to this axis (see arrows in Figure 3 b); the insert showing the indicatrix confirms the orientation of the NLC effect relative to the molecular arrangement.

**Figure 3:**
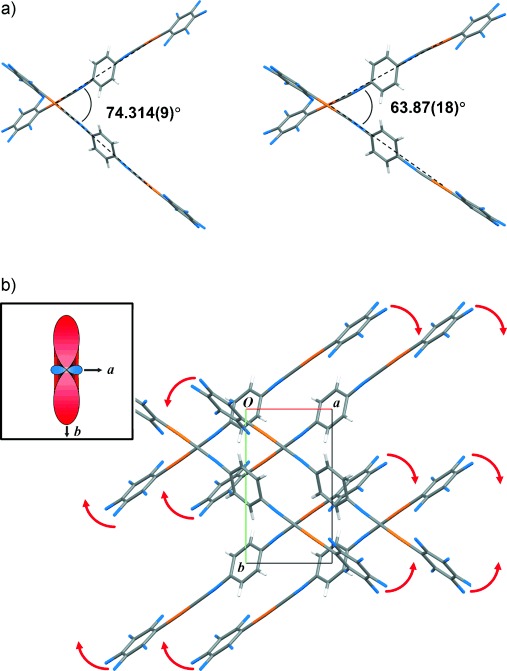
a) Torsion angle between molecules of separate layers, measured through the gold atoms at ambient pressure and 2.42 GPa. b) Molecular re-arrangement associated with increasing pressure in [(C_6_F_5_Au)_2_(μ-1,4-diisocyanobenzene)] viewed down the *c* axis. Inset: Cross-section of the indicatrix relative to the crystal structure.

While the molecular reorientation in **1** that causes the extreme NLC effect has similarities to that observed in framework and molecular systems it differs significantly since the pivot point is not associated with an atomic center or a covalent bond. The process occurs by the reorientation of molecules between which there are no significant intermolecular interactions, and is dependent on the relative orientation of molecules within the crystallographic unit cell. The study demonstrates that molecules of an appropriate shape and packed in the correct manner is sufficient for NLC behavior to be observed in a material. Thus, other linear molecules could, with no strong intermolecular interactions, be good candidates for NLC materials.

Luminescence spectra (Figure 4 a) over the pressure range 0–5 GPa were recorded to investigate whether the previously observed mechanochromism could be correlated with the observed NLC effect. The spectra with maxima at about 650 nm at ambient pressure has previous been identified as a π–π* transition.7a A shift to lower energy is observed as pressure increases to about 700 nm at 2.90 GPa, equating to a peak shift of 440+/−20 cm^−1^ GPa^−1^, which is less than half the typical literature values for systems where aurophilic interactions are photophyiscally important.^11^ It may be concluded that the observed shift with increasing pressure is consistent with conformational changes within the molecules rather than changes in interauric distances. These observations are consistent with both crystallographic data and the work of Ito et al., where dramatic changes in emission have been attributed to the formation of aurophilic interactions in an amorphous state.7a No such changes were observed in our study with crystallinity retained throughout. The transition reported here is likely due to the rotation of the central arene ring out of the plane of the terminal pentafluoroarene rings, reducing conjugation along the molecular unit (Figure 4 c).

**Figure 4:**
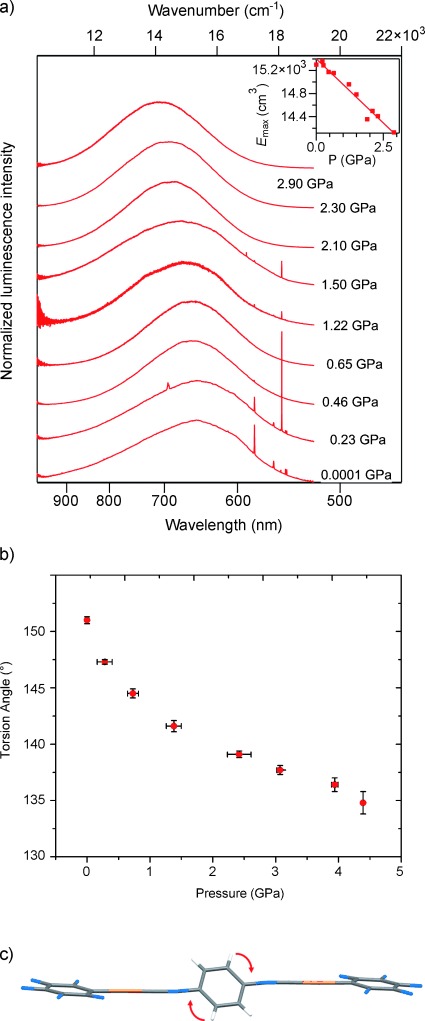
a) Luminescence spectra at variable pressure. Inset: energy of the luminescence band maxima. b) Torsion of the central arene ring relative to the plane of the molecule c) Direction of rotation of the central arene ring relative to the terminal pentafluorocyclopentene rings.

In conclusion, the mechanochromic complex [(C_6_F_5_Au)_2_(μ-1,4-Diisocyanobenzene)] **1** has been shown to display extreme NLC along the-*a* axis upon compression. The absence of any significant interactions or possible hinge mechanism in the structure makes clear the important role that molecular geometry, and crystal packing effects must play in creation of new materials that display extreme NLC. The work also demonstrates that despite the unique mechanochromic behavior, hydrostatic pressure is not analogous to mechanical pressure in inducing changes in emission wavelength, confirming the suggestion by Ito et al. that mechanically induced structural damage rather than pressure is responsible for the mechanochromic properties observed previously.

## Experimental Section

The compound was synthesized by following the procedure reported previously.7a All chemicals were used as received from the supplier and where required reactions were performed under nitrogen using Schlenk techniques.

High-pressure single-crystal X-ray diffraction experiments were performed on a 3-circle Bruker APEX II CCD diffractometer at station 11.3.1, Advanced Light Source, Lawrence Berkeley National Labs, US. A Merrill-Bassett diamond anvil cell was used for the high-pressure measurements using boehler-almax diamonds with 600 μm culets. Laser cut steel (250 μm thickness) was used as gasket material. Gasket holes were drilled using an Oxford Lasers laser mill to 200 μm.

Loading of the cell was performed using 4:1 methanol, ethanol mixture as a hydrostatic medium, and using ruby powder as the pressure calibrant. The pressure was monitored by ruby fluorescence stimulated by a 100 W 447 nm diode laser, measured by a fiber-optic coupled to a Princeton Instruments Acton 300i spectrometer.^8^

High-pressure data was integrated using the APEX 2 software suite. Shielding of the diffraction pattern by the DAC was dealt with by the generation of dynamic masks using an external program. Datasets were merged using XPREP and a multi-scan absorption correction was performed using SADABS.^12^ Data was refined against a previously determined room temperature structure by full-matrix least squares on *F* ^2^ using SHELXL-97.^13^ All C–C or C–N distances in the structure were restrained to the values of the room temperature structure on the assumption that such interactions are relatively resilient to compression. No such restraints were applied to metal–carbon interactions. The program Crystals was also utilized during the process of data analysis to identify and remove anomalous reflections.^14^ Features of WinGX, Olex-2, Xseed, Mercury, and CrystalExplorer were also used in the data analysis.^15^

CCDC 915028 http://www.ccdc.cam.ac.uk/cgi-bin/catreq.cgi–915035 http://www.ccdc.cam.ac.uk/cgi-bin/catreq.cgi contains the supplementary crystallographic data for this paper (structures for eight structure determinations carried out over the pressure range from ambient to 4.39 GPa). These data can be obtained free of charge from The Cambridge Crystallographic Data Centre via www.ccdc.cam.ac.uk/data_request/cif.

All equations of state were fitted using the program EOSFit.^9^ Principal axes of compressibility were calculated using PASCal.^16^

Luminescence spectra were measured with a Renishaw 3000 Raman imaging microscope equipped with a Peltier-cooled CCD camera. The excitation source was a 514 nm argon ion laser for the luminescence experiments. The microscope was used to focus light onto a spot of approximately 1 μm in diameter and to collect the scattered light. Pressure-dependent measurements on solid samples in nujol were made with a diamond-anvil cell (DAC, High-Pressure Diamond Optics). The ruby R1 line method was used to calibrate the hydrostatic pressure inside the gasketed cell. All pressure-induced phenomena reported here are reversible upon gradual release of external pressure.

## Notes

As a service to our authors and readers, this journal provides supporting information supplied by the authors. Such materials are peer reviewed and may be re-organized for online delivery, but are not copy-edited or typeset. Technical support issues arising from supporting information (other than missing files) should be addressed to the authors.
