# Supplementary material for: Hingeless Negative Linear Compression in the Mechanochromic Gold Complex [(C6F5Au)2(μ-1,4-diisocyanobenzene)]
Source: Angew Chem Int Ed Engl. 2013 Aug 2;52(37):9691–4. doi: 10.1002/anie.201302825 (PMC3795448; doi:10.1002/anie.201302825)
Supplement: Supplementary file 1 [file anie0052-9691-sd1.pdf]

Angewandte Chemie
Eine Zeitschrift der Gesellschaft Deutscher Chemiker

Supporting Information
© Wiley-VCH 2013
69451 Weinheim, Germany

Hingeless Negative Linear Compression in the Mechanochromic Gold Complex $[(C_6F_5Au)_2(\mu\text{-}1,4\text{-diisocyanobenzene})]$**

Christopher H. Woodall, Christine M. Beavers, Jeppe Christensen, Lauren E. Hatcher, Mourad Intissar, Andrew Parlett, Simon J. Teat, Christian Reber, and Paul R. Raithby**

anie_201302825_sm_miscellaneous_information.pdf

Supporting Information

Table S1. Crystallographic Parameters

Pressure (GPa)	ambient	0.28	0.73	1.38
**Formula**	$C_{20}H_4A_2F_{10}N_2$	$C_{20}H_4A_2F_{10}N_2$	$C_{20}H_4A_2F_{10}N_2$	$C_{20}H_4A_2F_{10}N_2$
**M_r**	856.19	856.19	856.19	856.19
**Crystal System**	Monoclinic	Monoclinic	Monoclinic	Monoclinic
**Space Group**	$P2_1/c$	$P2_1/c$	$P2_1/c$	$P2_1/c$
**a,b,c (Å)**	5.4580(6)<br>9.7540(11)<br>18.838(2)	5.5274(11)<br>9.4838(19)<br>18.410(4)	5.5924(11)<br>9.2665(19)<br>17.985(4)	5.6422(11)<br>9.0462(18)<br>17.618(4)
**β (°)**	91.6320(15)	92.32(3)	92.91(3)	93.39(3)
**V ($Å^3$)**	1002.5(2)	964.3(3)	930.8(3)	897.6(3)
**Z/Z’**	2/ 0.5	2/ 0.5	2/ 0.5	2/ 0.5
**ρ_{calc} / g cm^{-3}**	2.836	2.949	3.055	3.168
**Goodness of fit on F^2**	1.085	1.088	1.149	1.398
**Final R indices [I>2σ(I)]**	R_1= 0.0328<br>wR_2 = 0.0838	0.0369<br>0.1017	0.0358<br>0.1318	0.0444<br>0.1503
**Final R indices [All data]**	R_1= 0.0421<br>wR_2 = 0.0892	0.0450<br>0.1101	0.0442<br>0.1453	0.0566<br>0.1503
**$\Delta\rho_{max}$,$\Delta\rho_{min}$ (e $Å^{-3}$)**	0.556 / -0.646	0.686 / -0.807	0.920 / -0.904	1.204 / -1.298
**CCDC**	915028	915029	915030	915031

Pressure (GPa)	2.42	3.07	4.39
**Formula**	$C_{20}H_4A_2F_{10}N_2$	$C_{20}H_4A_2F_{10}N_2$	$C_{20}H_4A_2F_{10}N_2$
**M_r**	856.19	856.19	856.19
**Crystal System**	Monoclinic	Monoclinic	Monoclinic
**Space Group**	$P2_1/c$	$P2_1/c$	$P2_1/c$
**a,b,c (Å)**	5.6829(11)<br>8.7611(18)<br>17.176(3)	5.6826(11)<br>8.6775(17)<br>17.010(3)	5.6400(11)<br>8.5223(17)<br>16.774(3)
**β (°)**	94.06(3)	94.40(3)	95.12(3)
**V ($Å^3$)**	853.0(3)	836.3(3)	803.0(3)
**Z/Z’**	2/ 0.5	2/ 0.5	2/ 0.5
**ρ_{calc} / g cm^{-3}**	3.333	3.400	3.493
**Goodness of fit on F^2**	1.254	1.030	1.425
**Final R indices [I>2σ(I)]**	R_1= 0.0391<br>wR_2 = 0.1422	0.0317<br>0.0735	0.0549<br>0.1804
**Final R indices [All data]**	R_1= 0.0421<br>wR_2 = 0.1422	0.0393<br>0.0766	0.0718<br>0.1882
**$\Delta\rho_{max}$,$\Delta\rho_{min}$ (e $Å^{-3}$)**	0.911 / -0.861	1.115 / -0.846	1.962 / -1.850
**CCDC**	915033	915034	915035

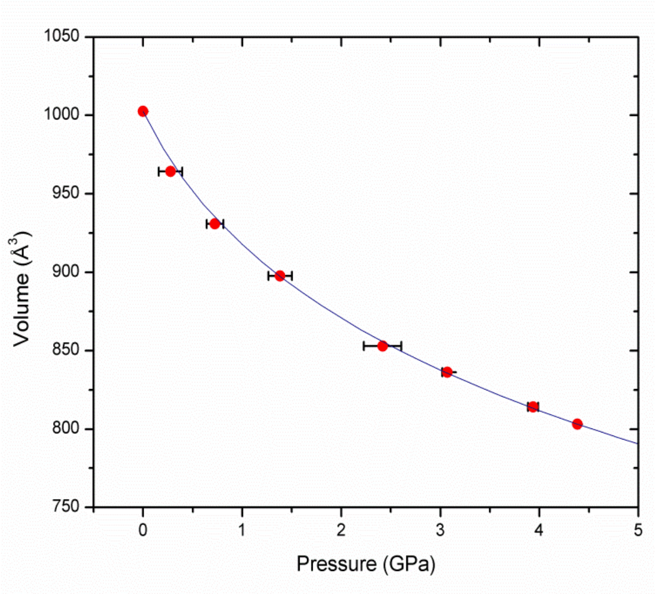

Figure S1 Unit cell volume as a function of pressure. Error bars on pressure measurements are given. A 3rd order Birch-Murnaghan equation of state has been plotted

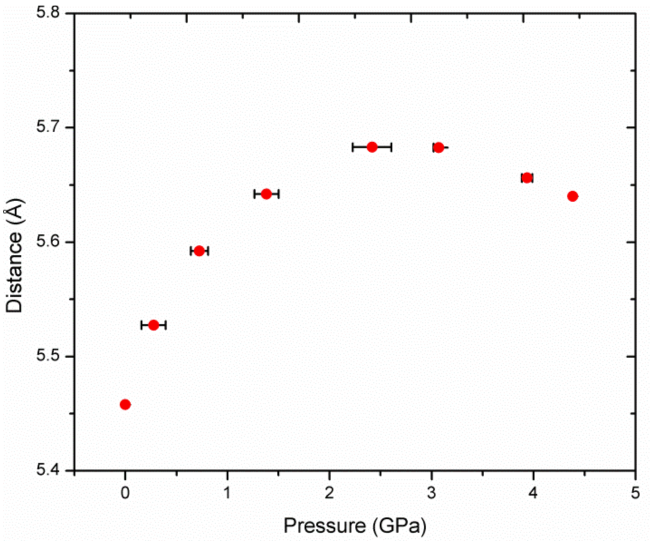

Figure S2.Change in a axis length with pressure

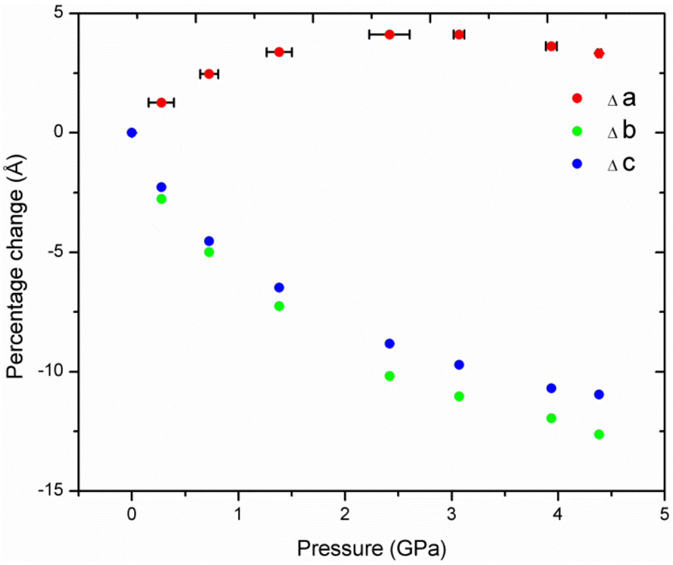

Figure S3. Change in crystallographic parameters with pressure

Table S2. Results of equation of state calculations from EOSFit.[1]

Equation fitted	Parameter					
	V_0	K_0	K'	K''	X^2w	$delP_{max}$
**Murnaghan**	1002.51(19)	8.54(51)	6.75(45)	-	0.7488	-0.103
**Birch Murnaghan 2nd order**	1002.46(60)	12.57(16)	4	-	9.1901	-0.386
**Birch Murnaghan 3rd order***	1002.51(20)	7.49(70)	9.80(1.3)	-5.78	0.4226	-0.082
**Birch Murnaghan 4th order**	1002.52(19)	5.28(1.94)	21.46(13.94)	-75.40(138.11)	0.1547	-0.103

The third order Birch Murnaghan was selected for use due to the low X^2w value and acceptable esd size.

Table S3. Details of principal axis strain calculations from PASCal up to 2.41 GPa.[2]

Axes of compresssion			Direction		
	K(TPa^{-1})	σK(TPa^{-1})	a	b	c
**X1**	31.3453	0.4196	0	1	0
**X2**	29.3314	2.4840	0.4866	0	0.8736
**X3**	-12.5703	2.5099	0.9993	0	-0.0384
**V**	61.0274	6.5625			

Table S4. Details of principal axis strain calculations from PASCal for all observed data.[2]

Axes of compresssion			Direction		
	K(TPa^{-1})	σK(TPa^{-1})	a	b	c
**X1**	16.6126	2.1649	0	-1	0
**X2**	15.5703	1.9531	0.4772	0	0.8788
**X3**	-4.1651	3.9998	0.9993	0	-0.0365
**V**	44.6175	4.2206			

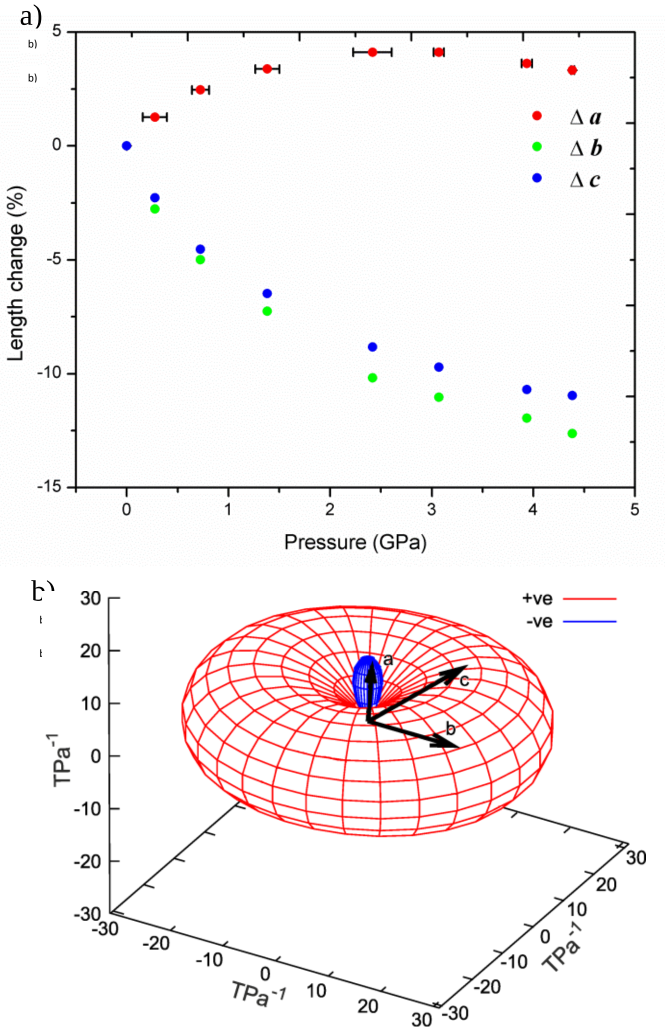

Figure S4. a) Change in cell dimension length with pressure as a percentage of ambient length. b) The compressibility indicatrix of **1** relating the principal axes of compression (red = positive; blue = negative compression) to the crystallographic axes (black). Notice the negative compressibility along the *a* axis.

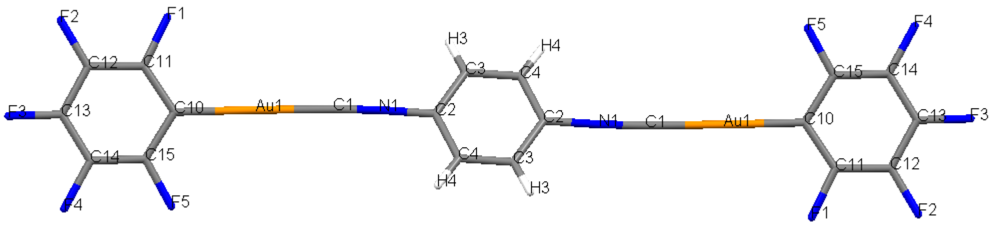

Figure S5. Labelled molecular structure of [(C_6F_5Au)$_2$(μ-1,4-Diisocyanobenzene)]

Table S6. Intramolecular distances and angles of interest

Pressure (GPa)	Distance (Å)				Torsion Angle (°)
	C10-Au1	C1-Au1	C1-N1	C2-N2	(C10-C16) centroid to (C2-C3)
**ambient**	2.017(5)	1.953(5)	1.146(7)	1.393(7)	151.0(2)
**0.28**	2.005(4)	1.943(6)	1.152(7)	1.409(6)	147.3(2)
**0.73**	2.008(8)	1.932(9)	1.156(10)	1.402(9)	144.52(6)
**1.38**	2.014(10)	1.961(11)	1.137(12)	1.412(11)	141.6(5)
**2.42**	2.013(14)	1.980(18)	1.104(17)	1.408(15)	139.1(3)
**3.07**	2.000(7)	1.948(8)	1.146(10)	1.399(9)	137.7(4)
**3.94**	2.003(19)	1.919(14)	1.162(14)	1.372(13)	136.4(6)
**4.39**	1.956(19)	1.929(18)	1.143(16)	1.393(15)	134.8(10)

Table S7. Intermolecular distances and angles of interest

Pressure (GPa)	Distance (Å)			Torsion Angle (°)
	Au1-Au1	(C10-C16) centroid to Au1	(C10-C16) plane to Au1	Golds in *ac* layer
**ambient**	5.4580(6)	3.554(7)	4.1301(4)	74.310(9)
**0.28**	5.5274(11)	3.306(4)	3.587(2)	71.295(16)
**0.73**	5.5924(11)	3.34(2)	3.8915(10)	68.69(2)
**1.38**	5.6422(11)	3.196(18)	3.476(16)	66.375(17)
**2.42**	5.6829(11)	3.121(9)	3.6774(7)	63.87(18)
**3.07**	5.6826(11)	3.104(11)	3.546(9)	63.301(16)
**3.94**	5.6562(11)	3.011(15)	3.6261(8)	63.24(19)
**4.39**	5.6400(11)	2.97(3)	3.6109(12)	63.14(3)

1. Angel, R., *EOSFIT version 5.2*. 2002.
2. Cliffe, M.J. and A.L. Goodwin, *PASCal: a principal axis strain calculator for thermal expansion and compressibility determination.* Journal of Applied Crystallography, 2012. **45**: p. 1321-1329.
